# Experimental models of liver-stage malaria: Progress, gaps, and challenges

**DOI:** 10.1371/journal.ppat.1013796

**Published:** 2025-12-18

**Authors:** Norapat Nitaramorn, Kasem Kulkeaw, Mallika Imwong

**Affiliations:** 1 Biodesign in Medicine Program, Graduate School, Mahidol University, Nakhon Pathom, Thailand; 2 Siriraj Integrative Center for Neglected Parasitic Diseases, Department of Parasitology, Faculty of Medicine of Siriraj Hospital, Mahidol University, Bangkok, Thailand; 3 Siriraj-Long Read Laboratory, Faculty of Medicine Siriraj Hospital, Mahidol University, Bangkok, Thailand; 4 Department of Molecular Tropical Medicine and Genetics, Faculty of Tropical Medicine, Mahidol University, Bangkok, Thailand; Institut Pasteur, FRANCE

## Abstract

For decades, achieving malaria eradication has proven difficult. *Plasmodium* parasites first multiply asymptomatically in the liver before causing cyclic erythrocytic infections and clinical symptoms. Unlike other species, *Plasmodium vivax* can remain dormant for months or years as hypnozoites within hepatocytes. When these latent parasites reactivate, they cause clinical episodes in most *vivax* malaria cases. Thus, targeting the liver stage is essential to prevent disease progression and relapse. Current therapies are effective but pose risks for individuals with glucose-6-phosphate dehydrogenase deficiency. Consequently, developing and evaluating new antimalarial agents requires *in vitro* models that accurately represent intrahepatic parasite growth. Existing *in vitro* models have improved over time, but still face limitations. Recent advancements in cell culture have introduced organoids, which are three-dimensional, self-organizing cellular structures that recreate the microarchitecture and functions of native tissues. This review highlights the strengths and weaknesses of current experimental platforms and identifies critical gaps. We discuss how organoid technology can address these shortcomings, guiding improvements in liver-stage malaria models. Such advances will facilitate the development of preventive strategies and radical cures.

## Author summary

Ethical concerns regarding animal use and advances in cell culture have driven major improvements in *in vitro* disease models. Although often neglected, the liver stage is crucial for preventing and eradicating malaria. Modeling *Plasmodium* development in hepatocytes is more complex than modeling blood-stage parasites because preserving hepatocyte functions in cell culture is challenging. This review examines the progress and ongoing challenges associated with using three-dimensional cell culture systems to study liver-stage malaria.

## 1. Introduction

Since Laveran’s discovery of blood parasites in 1880 [1,2], malaria has threatened millions of lives worldwide [2]. Malaria eradication is hindered by drug resistance and persistent latent infections in the liver, causing multiple relapses. Current therapies prevent clinical disease and relapse but raise safety concerns and show limited coherence. Only two drugs, primaquine and tafenoquine, have been available for over two decades, serving as prophylaxis and radical cures. Both can induce red blood cell lysis in individuals with glucose-6-phosphate dehydrogenase deficiency, which limits large-scale use. Consequently, new drug candidates and improved *in vitro* models capable of representing both active and dormant parasites in hepatocytes are essential. This review examines the usefulness and gaps in existing experimental platforms. Emerging three-dimensional (3D) cell culture systems, including organoids, better recapitulate native tissue organization and function. We summarize the progress in 3D liver-stage malaria modeling and discuss potential enhancements to accelerate drug discovery and support eradication efforts.

## 2. Challenges in malaria eradication

**Fig 1:**
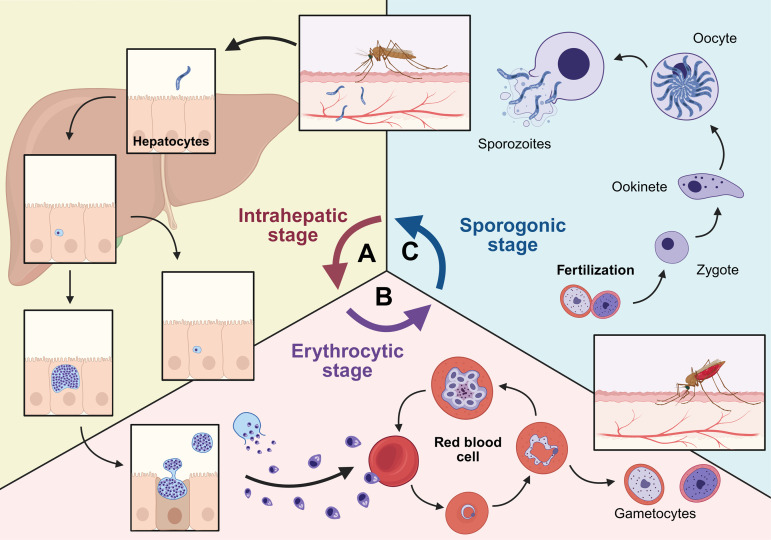
Cyclic development of *Plasmodium* species in three distinct niches of hosts. In mammalian hosts, asexual development of all *Plasmodium* species begins in the liver. **(A) Exo-erythrocytic (liver-stage) development:** Following a blood meal, sporozoites are released from the salivary glands of a female *Anopheles* mosquito into the skin. They travel through blood vessels and invade hepatocytes. Inside the hepatocytes, sporozoites undergo multiple rounds of cell division—schizogony—to produce schizonts. In the case of *P. vivax* and *P. ovale*, some sporozoites enter a dormant, non-proliferative stage called a hypnozoite. Once schizonts fully mature, merosomes egress from the infected hepatocytes into the bloodstream. **(B) Erythrocyte-stage development:** Merozoites invade red blood cells and undergo schizogony to generate additional merozoites. Some ring trophozoite stages differentiate into male and female gametocytes, which are transmitted to a female *Anopheles* mosquito during a subsequent blood meal. **(C) Sporogony-stage development:** In the mosquito midgut, ingested gametocytes fertilize to form zygotes. These develop into ookinetes and then oocysts, which produce numerous sporozoites. The sporozoites migrate to the mosquito’s salivary glands and await transmission to a human host at the next blood meal. Created in BioRender. Kulkeaw, K. (2025) https://BioRender.com/qbcziwc.

*Plasmodium* species are obligate intracellular protozoans that infect diverse vertebrates, including lizards, birds, and mammals. Five species infect humans: *P. falciparum*, *P. vivax*, *P. ovale*, *P. malariae*, and zoonotic *P. knowlesi,* and have complex life cycles (Fig 1). Nearly half of all countries remain malaria endemic, and about one-quarter of the global population is at risk, with over 200 million infections each year [3]. In 2015, the World Health Organization (WHO) deployed the Global Technical Strategy for Malaria. The strategy aimed to reduce malaria incidence and mortality by 90% between 2016 and 2030 [3,4]. While WHO-led campaigns achieved substantial reductions in global incidence between 2000 and 2019, progress has stalled. Annual cases plateaued at approximately 252 million from 2020 to 2023, highlighting the formidable challenges in achieving eradication [5].

Among the five major *Plasmodium* species infecting humans, *P. falciparum* malaria exhibits the highest prevalence, followed by *P. vivax*. *P. falciparum* occurs predominantly in Africa, whereas *P. vivax* is widespread across continents. Infections with *P. falciparum* can cause severe clinical manifestations, posing a life-threatening risk, especially to young children with limited immunity. Despite substantial efforts toward developing a protective vaccine targeting infective sporozoites, controlling *P. vivax* remains more challenging due to its latent liver-stage forms. *P. vivax* hypnozoites, which are non-proliferative, can persist in hepatocytes for months or years and may unexpectedly reinitiate cell division. Their reactivation leads to multiple relapses *in vivax* malaria [6–8]. These dormant stages remain undetectable with current laboratory tools. Most vivax malaria episodes result from hypnozoite reactivation. Hypnozoite reservoirs often evade immunity, remain asymptomatic, and maintain low parasitemia. Notably, *P. vivax* sexual stages can be transmitted from humans to Anopheles mosquitoes before symptoms arise. Thus, repeated relapses sustain malaria transmission [9] and exacerbate morbidity, particularly in infants, children, and pregnant women [10].

The updated Global Technical Strategy for Malaria (2016–2030) placed *P. vivax* hypnozoite reservoirs as a strategic target to achieve malaria-free status [10,11]. Mass administration of anti-hypnozoite drugs underlies these efforts. However, only two drugs-primaquine and tafenoquine, both 8-aminoquinolines-effectively eliminate *P. vivax* hypnozoites. These drugs can cause hemolytic anemia in individuals deficient in glucose-6-phosphate dehydrogenase (G6PD). Thus, G6PD deficiency testing is mandatory before administration, consuming additional resources and time. Moreover, these drugs are contraindicated for infants, pregnant women, and breastfeeding mothers, limiting large-scale implementation [11,12]. The urgent need to discover safer anti-relapse therapies targeting *P. vivax* hypnozoites is clear. Advancing our understanding of liver-stage malaria, including its molecular and cellular mechanisms, will facilitate the development of improved *in vitro* disease models.

## 3. Utility and drawbacks of the current liver-stage malaria models

*In vivo* and *in vitro* disease models have clarified many molecular and cellular mechanisms of sporozoite invasion and schizogony. Cancerous or immortalized cell lines and primary human hepatocytes often serve as *in vitro* models. Humanized rodent models provide an *in vivo*—relevant setting but still lack immune cells and human vasculature. Recognizing similarities and differences among these models is crucial for choosing the most appropriate platform for a given study.

### 4.1 Hepatic and non-hepatic cell lines

**Fig 2:**
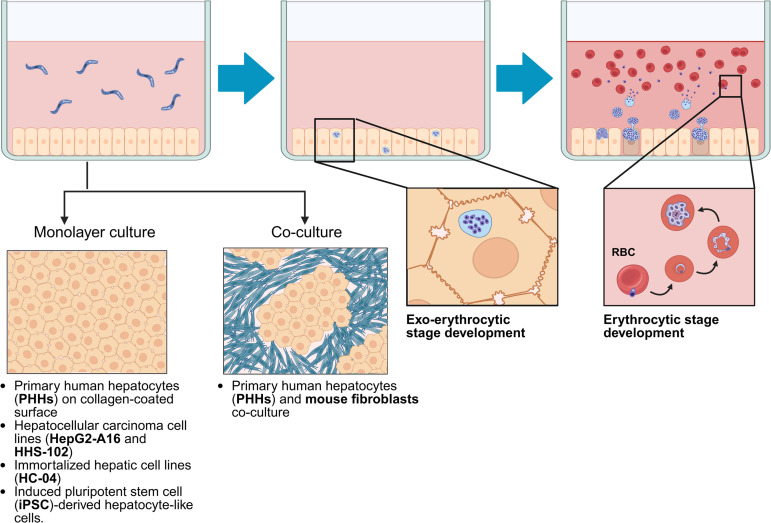
Two-dimensional (2D) cell culture systems for modeling liver-stage malaria. To study liver-stage development of *Plasmodium* spp., several human hepatic cell types are used: primary human hepatocytes (PHHs), hepatocellular carcinoma lines (e.g., HepG2-A16 and HHS-102), immortalized hepatic cell lines (e.g., HC-04), and induced pluripotent stem cell (iPSC)-derived hepatocyte-like cells. Hepatocellular carcinoma and immortalized cells typically grow as monolayers on rigid plastic surfaces. In contrast, PHHs must be cultured on collagen type IV-coated surfaces or co-cultured with murine fibroblasts. Sporozoites are introduced into the culture well, often followed by centrifugation or reduced medium volume to enhance sporozoite-cell contact. Successful liver-stage development is commonly indicated by segmented schizonts, which serve as a proxy for parasite development. Detection of free merozoites in the culture medium is less frequent in most available liver-stage assays. Created in BioRender. Kulkeaw, K. (2025) https://BioRender.com/3mgz0tq.

Cancerous cell lines, such as hepatocellular carcinoma lines, are commonly used due to simpler laboratory protocols (Fig 2). HepG2-A16, a HepG2 subclone, supports initial *P. vivax* sporozoite infection and schizogony [13,14]. In 1985, Hollingdale et al. first observed two intracellular forms of *P. vivax* in HepG2-A16 cells: small and large intrahepatic forms [13]. The small forms persisted, while the large schizonts eventually disappeared. In 2010, Chattopadhyay et al. used HepG2-A16 cells to test primaquine efficacy. The hepatic infection rates were 1.8% and 0.9% on days 3 and 9 post-infection, respectively. After three days of primaquine exposure, hepatic schizonts reduced by up to 40%. However, neither study confirmed complete intrahepatic development because detection relied on erythrocyte-infectious merozoites. Detecting *P. vivax* merozoites is more complex than detecting *P. falciparum* due to the need for human reticulocytes. The small *P. vivax* intrahepatic form is presumed to be a hypnozoite, but its reactivation has not been tested in HepG2-A16 cells. A major limitation of HepG2-A16 cells is their tendency to detach from cell culture surfaces, likely due to uncontrolled cancer cell growth. This instability complicates long-term culture required to study hypnozoite formation and reactivation. Moreover, HepG2-A16 cells have lower cytochrome enzyme activity than primary human hepatocytes [15], preventing primaquine bioactivation and possibly explaining the low observed efficacy [15]. In addition to the HepG2-A16 cell line, another hepatoma-derived cell line, HHS-102, exhibits characteristics more similar to normal human hepatocytes. Unlike HepG2 cells, HHS-102 cells can form bile canaliculi and halt their growth upon reaching confluence, thereby preventing overgrowth and detachment [16,17]. When these cells were tested for the intrahepatic development of *P. falciparum*, ring-stage parasites were observed in human erythrocytes, indicating that complete schizogony had occurred.

The human Huh7 cell line originates from hepatoma cells. Although *P. berghei* naturally infects rodents, Huh7 cells also permit *P. berghei* infection. Using Huh7 cells has advanced our understanding of sporozoite invasion and productive infection. A study of *P. berghei* in Huh7 cells highlights membrane ruffles as crucial for sporozoite invasion [18]. Live cell imaging shows that membrane ruffles and filopodia extensions increase during invasion. Rho GTPase, a regulator of plasma membrane dynamics, is identified as a required host factor. Additionally, *P. berghei* sporozoite traversal triggers membrane ruffling. Treating Huh7 cells with a membrane ruffling inhibitor decreases the percentage of intrahepatic schizonts, suggesting reduced productive invasion. For complete schizogony, multidrug resistance-associated protein is essential for forming mature *P. berghei* schizonts in Huh7 cells [19]. *P. berghei* also hijacks host Golgi in Huh7 cells to complete intrahepatic development, confirmed in primary human hepatocyte cultures [20]. Furthermore, the parasite uses a nutrient transporter to establish the parasitophorous vacuole and maintain parasite load in Huh7 cells [21]. Huh7 cells also serve as a drug testing platform. For example, an antiretroviral drug used for HIV therapy impairs *P. berghei* development in Huh7 cells [22]. Genomic analysis indicates that the parasite relies on protein trafficking pathways. In the *P. berghei*-infected Huh7 model, knocking down genes involved in protein trafficking reduces schizont size but not infection rate [23].

Because HepG2 cells lack CD81, a key circumsporozoite protein (CSP)-binding receptor for *P. falciparum* sporozoite invasion [24], primary human hepatocytes were immortalized to generate the HC-04 cell line. HC-04 cells express albumin, transferrin, and other liver proteins under standard conditions [25]. They support complete schizogony of *P. falciparum* and *P. vivax*, as indicated by the presence of liver schizonts. Additionally, the HC-04.J7 cell line, a subclone of HC-04, shows higher infection efficiency. By limiting dilution, HC-04 subclones were obtained and screened for *P. falciparum* infectivity. The HC-04.J7 line had an infection rate threefold higher than HC-04. Comparing the transcriptome and proteome of HepG2, HC-04, and HC-04.J7 cells identified glypican-3 as a potential receptor for *P. falciparum* sporozoites [26]. Glypican-3 occurs only in HC-04 and HC-04.J7 cells, not in HepG2, suggesting HepG2’s lower susceptibility to *P. falciparum*. The HC-04.J7 line was later used as a sporozoite infectivity assay for anti-CSP monoclonal antibodies [18]. However, both HC-04 and HC-04.J7 proliferate rapidly, rendering them unsuitable for long-term studies, including hypnozoite formation and reactivation, due to eventual overgrowth and detachment.

To overcome the rapid proliferation issue, a mesenchymal-cell-derived immortalized hepatocyte-like cell line (imHC) was established, exhibiting a lower proliferation rate than HC-04 cells. The sporozoite infection rate in imHCs is comparable to that in HC-04 cells. The detection of parasitized erythrocytes in imHC cultures confirmed the production of merozoites, indicating that *P. vivax* undergoes complete hepatic schizogony in this system [27]. Despite the advantages of these cancer-derived or immortalized hepatic cell lines, they have inherent limitations. The uncontrolled growth of cancer cells often leads to detachment from the culture surface, complicating long-term maintenance and thus hindering studies of hypnozoite reactivation. Furthermore, the hepatic functions of cancer cells are generally reduced compared to mature hepatocytes. HepG2 cells, for instance, show lower transcript levels of most cytochrome enzymes than primary human hepatocytes [28], and lipid accumulation in hepatoma lines is lower than in primary human hepatocytes [29].

For non-hepatic cell lines, HeLa cells are epithelial carcinoma cells derived from human cervical cancer. They have been widely used to investigate fundamental cell biology. Despite not originating from hepatocytes, human HeLa cells are susceptible to *P. berghei* infection and contribute to liver-stage malaria research. Initially, HeLa cells helped reveal SPECT as a parasite factor for *P. berghei* cell traversal [30,31]. They also demonstrated how *P. berghei* exploits autophagy to survive [32]. HeLa cell cultures are not limited to studying host and parasite factors alone. Using transgenic *P. berghei* with fluorescent proteins, researchers identified stage-specific parasite transcripts from early to late schizonts in HeLa cells [33]. The resulting transcriptome database is accessible at the European Nucleotide Archive (http://www.ebi.ac.uk/ena/) under accession numbers PRJEB23770 and ERP105548. Finally, *P. berghei* parasites utilize host Golgi for complete intrahepatic schizogony in HeLa cells, echoing findings in Huh7 and primary human hepatocytes [20].

### 4.2 Primary human hepatocytes

Primary human hepatocytes (PHHs) can be isolated from adult donor liver biopsies or whole donated livers. Under optimal culture conditions, PHHs retain hepatic functions for approximately 1–2 weeks. Micropatterned co-cultures with fibroblasts extend these functions beyond 3 weeks, enabling studies of HCV, HBV, and *Plasmodium* (including *P. falciparum* and *P. vivax*) [34–36] (Fig 2). PHHs support the intrahepatic development of *P. vivax*, with schizonts visible around 5–6 days post-infection. Adding human reticulocytes at days 12–14 post-infection enables the detection of erythrocyte-infective merozoites, confirming complete schizogony [37]. Unlike cancerous or immortalized cell lines, PHH culture is more complex and expensive, often requiring growth factors, hormones, and a collagen layer for cell adherence. PHHs also tend to dedifferentiate over time, losing their hepatic functions. Coculturing PHHs with fibroblasts can mitigate dedifferentiation [35,38]. While long-term cultivation can be achieved, the infection rate varies significantly depending on the donor. Thus, prescreening frozen PHH batches to ensure adequate infection rates is often necessary before schizogony studies or drug testing [39]. In addition, a limited supply of PHHs and challenges in scaling them up pose constraints for large-scale drug screening. Using smaller culture wells can reduce the number of PHHs required and facilitate high-throughput drug testing [40]. Microscale cultures of PHHs combined with high-content imaging technologies have been shown to overcome these limitations, enabling efficient large-scale screening of antimalarial compounds [35,38,40].

### 4.3 Primary porcine hepatocytes

Parasite–host selectivity is commonly acknowledged. However, rodent *P. yoelii* sporozoites can infect and develop in both human and mouse hepatocytes, whereas *P. falciparum* sporozoites cannot invade mouse hepatocytes [24]. Intriguingly, *P. falciparum* sporozoites can infect porcine hepatocytes but exhibit atypical parasitophorous vacuoles, causing a mid-stage developmental arrest [41]. Thus, porcine hepatocytes are permissive to *P. falciparum* sporozoites but can’t support parasite development.

### 4.4 Induced pluripotent stem cell-derived hepatocyte-like cells (iPSC-derived HLCs)

Because PHHs are finite and non-expandable, induced pluripotent stem cells (iPSCs) provide a promising alternative source of functional hepatocytes (Fig 2). iPSCs, derived from various somatic cell types, can proliferate indefinitely under optimal conditions, maintain genetic stability, and differentiate into multiple cell lineages. This capability allows the large-scale production of hepatocytes suitable for high-throughput testing. Si-Tayeb et al. demonstrated that mouse embryonic fibroblast-derived iPSCs could differentiate through distinct stages—endoderm, hepatic endoderm, hepatoblasts, and then hepatocyte-like cells—mimicking embryonic tissue formation [42]. These iPSC-derived hepatocyte-like cells (HLCs) express hepatocyte markers such as HNF4a, AFP, and ALB, and perform functions similar to PHHs, including albumin secretion, glycogen and lipid accumulation, and metabolic activities.

A study utilizing iPSC-derived HLCs showed that these cells, which express CD81 and SR-B1 receptors necessary for parasitophorous vacuole formation, support the intrahepatic development of *P. falciparum* and *P. vivax* at infection rates of 0.003%–0.02% [43]. This platform is suitable for testing atovaquone, a drug that is active without metabolic conversion. By contrast, primaquine requires cytochrome P450 2D6 metabolism, and the reduced cytochrome P450 activity in iPSC-derived HLCs impedes proper primaquine testing. Studies indicate that iPSC-derived HLCs resemble fetal hepatocytes, with significantly lower levels of urea production, albumin secretion, and cytochrome P450 activity compared to PHHs [44,45]. These cells also express lower levels of hepatic drug-metabolizing enzymes than PHHs [46], reflecting an immature, fetal-like state. To address this, strategies involving small molecules or specialized culture plates are used to enhance cytochrome P450 activity and thus enable primaquine sensitivity testing. However, although a non-replicative hypnozoite form was observed in iPSC-derived HLCs, no erythrocyte-infected parasites were detected [43]. Improving hepatocyte maturity remains essential before iPSCs can serve as a reliable PHH substitute [46,47].

### 4.5 Humanized mouse models

Mice and rats are natural hosts of *P. yoelii* and *P. berghei*, but neither form hypnozoites [48]. To model human liver-stage *vivax* malaria, researchers used commercially available human liver-chimeric mice [49,50]. These humanized mice lack functional mouse hepatocytes due to the deletion of the fumarylacetoacetate hydrolase (*Fah*) gene. *Fah*-knockout mice suffer from hepatocellular damage caused by the accumulation of toxic intermediates. Transplanting human hepatocytes into these mice replaces the damaged mouse hepatocytes, thereby creating a platform to study human liver-stage malaria in a physiologically relevant setting. To prevent immune rejection of xenografts, a severe combined immunodeficient (SCID) mouse line that lacks T and B lymphocytes (*Rag2−/−IL2rg−/−*) is combined with *Fah−/−* mice, creating FRG mice [51].

*P. vivax* sporozoites are intravenously injected into human hepatocyte-chimeric FRG mice. Two intrahepatic forms appear: a large form containing multiple merozoites that actively replicate, and a small, non-replicating form persisting up to 21 days post-infection, presumed to be a hypnozoite. After intravenous injections of human reticulocytes, blood-stage *P. vivax* parasites appear on day 9 post-infection. By day 14, hypnozoite-like forms remain visible, whereas the fully developed schizonts disappear. By day 21, most intrahepatic parasites persist as putative hypnozoites, with some increasing in size, suggesting possible reactivation. Recent studies show hypnozoite reactivation via secondary schizont formation at later time points [52,53]. Using FRG mice, atovaquone inhibits intrahepatic schizogony, while primaquine acts as prophylaxis, preventing relapse. Drug efficacy is evaluated via immunofluorescence and *P. vivax* 18S rRNA-based PCR [49]. By backcrossing the FRG mouse onto a non-obese diabetic (NOD) strain, *Fah−/−Rag2−/−IL2rg−/−*NOD (FGRN) mice are generated. These mice have defective macrophages that cannot engulf human grafts [54,55], allowing the successful repopulation of human erythrocytes. In these FGRN mice, all blood-stage forms of *P. vivax*, including gametocytes, have been observed. This system enabled testing of monoclonal antibodies targeting *P. vivax* Duffy Binding Protein to inhibit human reticulocyte invasion [56–60].

### 4.6 Three-dimensional (3D) culture models

Advances in cell culture technology have led to the improvement of *in vitro* models that better replicate human tissue pathology. Conventional two-dimensional (2D) cultures differ significantly from actual tissues. Many three-dimensional (3D) culture platforms, involving single or multiple cell types interacting with matrix proteins, now exist. Cells can form spheroids—aggregates that resemble some aspects of native tissues—or, using stem cells, can self-organize into organ-like structures known as organoids. Mellin and Boddey previously discussed using organoids for malaria research, but relatively few studies have been published [61]. Here, we update current applications and potential improvements of spheroids and organoids for modeling liver-stage malaria.

#### 4.6.1 Hepatic spheroid models.

Hepatic spheroids can form by aggregation or proliferation of cancerous or immortalized hepatic cell lines. They may develop with or without scaffolds. A semi-solid gel scaffold, containing extracellular matrix proteins, supports cell adherence and nutrient diffusion, while scaffold-free methods rely on low-attachment surfaces to encourage cell aggregation. Both approaches produce spheroids that often develop hypoxic cores due to limited oxygen diffusion, creating zones of dead or unhealthy cells that mimic tumor-like conditions. Although spheroid use in infectious disease research is limited, these models are utilized for studying liver-stage malaria.

Chua and colleagues generated spheroids from primary simian and human hepatocytes using a disc-shaped, porous Cellusponge scaffold [62]. Compared to 2D collagen-coated cultures, simian hepatic spheroids produced higher concentrations of urea over 22 days, though urea levels gradually declined, indicating dedifferentiation over time. Immunofluorescence confirmed hepatocyte polarization, showing basolateral protein CD147 and apical protein MRP2 in human hepatic spheroids. A fluorescent substrate for esterase activity assessed membrane integrity and cell viability. Transcript levels of *CYP1A2* and *CYP3A4* in spheroids were similar to those in 2D human hepatocyte cultures.

These human hepatic spheroids expressed the sporozoite receptor CD81, a proposed host factor for hepatocyte infection. To improve infection rates, *P. vivax* or *P. cynomolgi* sporozoites were incubated with hepatocytes before forming 3D spheroids. In-suspension incubation with simian hepatocytes increased infection rates approximately threefold compared to direct inoculation into pre-formed 3D spheroids, suggesting that the tight spheroid structure may hinder sporozoite entry. In-suspension infection allowed *P. cynomolgi* to complete development, producing blood-stage parasites in simian reticulocytes. Applying this strategy to human hepatic spheroids increased *P. vivax* sporozoite infection rates compared to 2D cultures, but the release of infective intrahepatic merozoites was not observed. The compact spheroid structure may entrap merozoites inside. Dissociating spheroids into single cells or inducing cell lysis might release merozoites, enabling subsequent erythrocyte infection. However, this study lacks microscopic data to confirm the proper development of parasites. Whether the 3D Cellusponge-based spheroids support hypnozoite formation and reactivation remains unexplored.

#### 4.6.2 Liver organoid.

**Fig 3:**
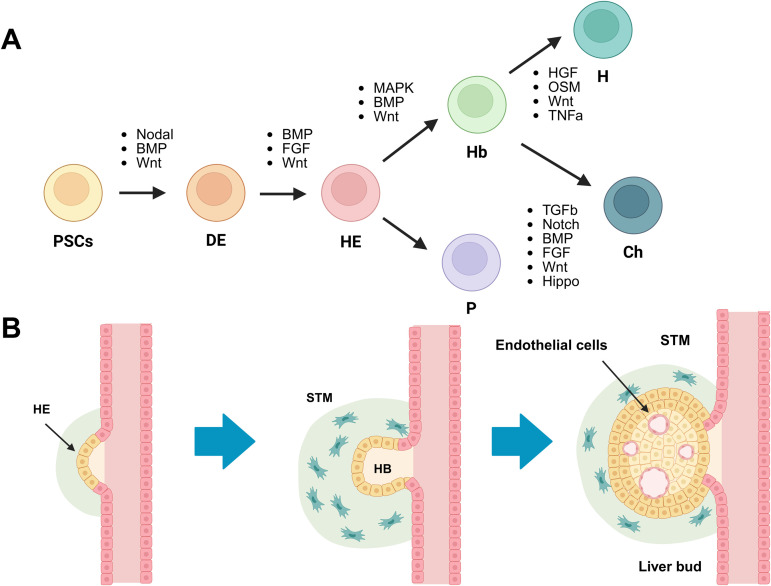
Liver development and differentiation of pluripotent stem cells (PSCs) into hepatocytes. Insights into liver development largely arise from mouse embryonic studies. Applying these principles to human PSCs, such as embryonic stem cells or iPSCs, reveals that external stimuli (e.g., growth factors, hormones) drive PSCs to differentiate into hepatocytes. **(A) External stimuli in hepatocyte generation from PSCs:** PSCs are first induced to form definitive endoderm (DE) using signals from Nodal, bone morphogenic protein (BMP), fibroblast growth factor-a (FGFa), and Wnt pathways. Once committed, DE differentiates into hepatic endoderm (HE), which can give rise to pancreatic cells (P), cholangiocytes (Ch), and hepatocytes (H). Distinct sets of stimuli direct HE to differentiate into either hepatocytes or cholangiocytes. **(B) Mouse liver budding:** In mouse embryos, the foregut endoderm, derived from DE, forms the liver, pancreas, and gall bladder. The hepatic endoderm (HE) migrates into the septum transversum mesenchyme (STM), where it differentiates into hepatoblasts and then hepatocytes. Hepatic and endothelial cell formation in STM creates a bud-like structure, resembling a developing liver bud. Created in BioRender. Kulkeaw, K. (2025) https://BioRender.com/nrtq41n.

Organoids arise from adult stem cells in a given tissue. Adult stem cells can divide and differentiate into mature, functioning cells to replace aged or damaged cells. Generally multipotent, these cells give rise to various specialized cell types. Under optimal biological stimuli, adult stem cells can self-organize and function in a manner similar to their tissue of origin. Organoids, three-dimensional structures formed from such stem cells, have emerged as valuable *in vitro* models to study tissue development and disease mechanisms [63,64]. Beyond multipotent adult stem cells, pluripotent stem cells can also be used to generate organoids (Fig 3A). Since organoid formation from both pluripotent and adult stem cells requires an understanding of embryogenesis and adult tissue maintenance, this section reviews current methods and assays for utilizing liver organoid platforms in the context of liver-stage malaria research, as well as their potential drawbacks.

***4.6.2.1 Hepatic organoids from adult and fetal liver:*** Although the presence of adult stem cells in the human liver is debated, isolating cell populations with stem cell-like properties has enabled the generation of human liver organoids. Leucine-rich-repeat-containing G-protein-coupled receptor 5 (Lgr5), a Wnt pathway target gene, is used as a marker of adult stem cells in the intestine, colon, and stomach. In mice, Lgr5 is normally undetectable in the liver but appears in bile duct epithelial cells following liver injury. These damage-induced, Lgr5-positive cells can form organoids in the presence of a Wnt activator [65]. Huch et al. isolated EpCAM-positive cells from human bile ducts and cultured them in a three-dimensional (3D) gel-based system, enabling the formation of hepatic organoids [65,66]. These differentiated liver organoids expressed hepatocyte markers (HNF4a, albumin, and cytochrome proteins), secreted albumin and bile acids, and exhibited CYP3A3/4/5 activities as well as phase I and II detoxifying functions. In addition to isolating stem cell-like fractions, hepatic organoids can also be established using hepatocytes directly. Organoids derived from human fetal liver tissue (11–20 weeks of gestation) and from cryopreserved primary human hepatocytes (PHHs) can be generated without additional cell isolation steps. Compared to two-dimensional (2D) PHH cultures and fetal liver-derived organoids, PHH-derived organoids show higher CYP3A4 activity. Albumin secretion levels remain similar among PHH-derived organoids, fetal-liver-derived organoids, and 2D PHH cultures [67].

Two recent studies have demonstrated the utility of fetal and adult liver organoids for modeling the intrahepatic development of *P. falciparum* [68,69]. When fetal liver organoids were adapted to a 2D culture platform, they supported *P. falciparum* schizogony, allowing detection of small and large intrahepatic forms with parasite-specific antibodies. However, erythrocyte infection by released merozoites remained difficult to observe. Single-cell transcriptomic analysis of these organoids revealed increased lipid and cholesterol synthesis, along with upregulation of Scavenger Receptor B1 (SR-B1), consistent with enhanced HDL-mediated lipid uptake.

Another study generated liver organoids from adult donor intrahepatic ductal cells (cholangiocytes) [65]. By embedding these dissociated cells in basement membrane extract, 3D organoids were formed. To introduce *P. falciparum* sporozoites, the organoid was dissociated into small fragments and coincubated with sporozoites for three hours before re-embedding in the 3D matrix. After six days, infection and parasite maturation were assessed using antibodies against *P. falciparum* HSP70 and merozoite surface protein 1. Infection rates varied among donors, with a maximum of approximately 0.8%. Maturation of intrahepatic forms was indicated by an increase in size, although infective merozoites capable of invading human erythrocytes were not detected. Despite this limitation, this platform offers a valuable *in vitro* assay for identifying novel drugs targeting the liver-stage development of *P. falciparum* [69].

***4.6.2.2 Hepatic organoids from iPSCs:*** Generating hepatic organoids from pluripotent stem cells (PSCs) is more complex than using adult stem cells, as it requires multiple lineage-specific differentiation steps that mirror embryonic hepatocyte development. After fertilization, the three germ layers—endoderm, mesoderm, and ectoderm—form. The endoderm gives rise to the gut tube, from which the liver develops. The hepatic endoderm migrates into the septum transversum mesenchyme (STM), ultimately differentiating into hepatoblasts and then hepatocytes. This process of hepatic endoderm migration and differentiation is called liver budding (Fig 3B). Maturation of the liver bud into a functional liver organ requires endothelial cells to establish proper vasculature.

iPSC-derived endodermal cells expressing EpCAM have been shown to form hepatic organoids resembling the fetal liver stage [70]. To achieve mature hepatocyte functions, an additional 10–14 days of *in vitro* culture are typically required. Our own experience confirms that iPSC-derived endoderm can also form hepatic organoids [47]. These organoids stably express the *Plasmodium* sporozoite receptor CD81 for up to 60 days, along with sustained albumin secretion and CYP4A3 expression. Moreover, intermediate endodermal cells obtained during the hepatic differentiation of human iPSCs in a 2D system can form spherical structures that, once embedded in Matrigel, gain mature hepatocyte functions, including CYP3A4 activity, urea production, and albumin secretion [71]. Whether these organoids also possess CYP3A4 and CYP2C19 activity necessary for primaquine metabolism remains to be investigated.

**Fig 4:**
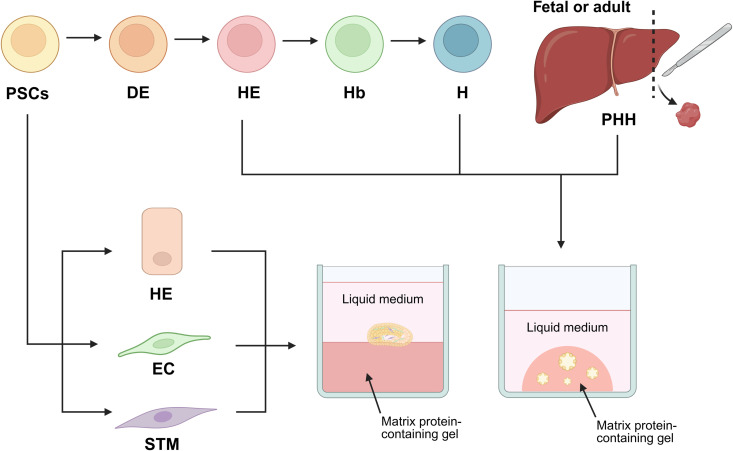
Methods for generation of liver organoids. Various methods produce liver organoids, depending on the cell source and 3D structure formation strategy. Currently, liver organoids can be generated from primary human hepatocytes (PHHs) or PSCs. PSC-derived hepatic endoderm (HE) or hepatocytes (H) can form 3D organoids through stages involving definitive endoderm (DE), HE, hepatoblast (Hb), and eventually H. PHHs may originate from fetal or adult livers. To create a 3D structure, cells are embedded in a gel-like scaffold, such as Matrigel (an extracellular matrix-based medium), then covered with liquid medium. Following the principles of mouse liver budding, combining HE, endothelial cells (EC), and STM on top of solidified Matrigel can produce a liver bud-like structure. Created in BioRender. Kulkeaw, **K.** (2025) https://BioRender.com/kkfupxg.

In 2013, researchers advanced the development of liver bud organoids by combining iPSC-derived hepatic endoderm with human umbilical vein endothelial cells and human mesenchymal stem cells [72]. Plating this three-cell mixture on Matrigel allowed self-organization into 3D cell clusters (Fig 4). Immunofluorescence staining showed clusters of AFP-expressing hepatoblasts surrounded by CD31-positive endothelial cells. Transplanting these liver bud organoids into severe combined immunodeficient (SCID) mice elevated human serum albumin levels in the host’s blood and improved survival in mice with induced hepatic injury. Thus, iPSC-derived liver bud organoids initially resemble fetal-stage liver *in vitro* but can acquire greater maturity upon *in vivo* engraftment. Recently, the protocol for hepatic organoid generation from human PSCs was further refined by optimizing the doses and exposure times of key factors [73].

The hepatic organoids can be stably expanded *in vitro* while preserving hepatocyte functionality. Using this revised protocol, Jin et al. demonstrated that PSC-derived hepatic organoids are more suitable for *in vitro* drug toxicity testing than conventional 2D PSC-derived hepatocyte cultures. Recently, researchers employed an iPSC-derived liver bud model to study liver-stage malaria. The human liver bud is generated from hepatic endoderm, septum transversum mesenchyme, and endothelial cells, all originating from human iPSC lines without requiring donated tissue. Immunofluorescence staining with anti-UIS4 revealed extremely low infection rates, detecting only a small parasite form. PCR-based analysis of the parasite burden failed to detect the *P. vivax* 18S rRNA transcript. Nevertheless, evidence for complete schizogony arises from the detection of blood-stage parasites in human reticulocytes [74].

When comparing recent studies using human fetal and adult liver organoids, sporozoite infection methods vary. Yang et al. plated fetal liver organoids as 2D monolayers [68], whereas Rao et al. dissociated adult liver organoids from a gel scaffold before incubating with *P. falciparum* sporozoites [69]. Similarly, Nitaramorn et al. incubated *P. vivax* sporozoites with 3D human liver buds extracted from a gel scaffold. Low infection rates may stem from low sporozoite penetration since the compact liver bud lacks vasculature. Interestingly, directly exposing *P. berghei* to human hepatic cell line-derived compact spheroids in a stirring tank enhances infection, implying that dynamic force is needed during sporozoite infection [75]. Despite low infection, iPSC-derived liver buds display altered lipid metabolism at the transcript level. After *P. falciparum* sporozoite invasion, transcripts involved in lipogenesis increase [68]. In *P. vivax*-infected human liver buds, the cholesterol synthesis gene TM7SF2 is upregulated, mirroring the findings of Yang and colleagues [74].

## 5. Key characteristics of the organoid system for modeling liver-stage malaria

An ideal *in vitro* disease model should closely recapitulate the *in vivo* microarchitecture and functions of human tissues. This section outlines key biological molecules that can improve *in vitro* liver-stage malaria models. The discussion is divided into two phases: pre-sporozoite incubation and post-sporozoite infection.

### 5.1 Pre-sporozoite incubation

Determining which cellular components are needed and their required characteristics will guide the design of improved organoid models. In the human body, immune cells extravasate by capturing, adhering, and transmigrating to damaged sites. Similarly, a key question for modeling liver-stage malaria is how sporozoites identify where to halt their migration across the endothelial barrier and enter the liver parenchyma to infect hepatocytes.

Sporozoite surfaces are rich in CSP and thrombospondin-related anonymous protein, and binding these proteins to their ligands is crucial [76–78]. Known ligands include highly sulfated heparan sulfate proteoglycans (HSPGs) [79] and low-density lipoprotein receptor-related proteins [80]. Hepatocyte invasion is triggered when CSP interacts with highly sulfated HSPGs, activating calcium-dependent protein kinase 6 and inducing the invasive phenotype of the sporozoites. CSP-HSPG binding also leads to the release of P36 and P52 proteins, which are required for forming the parasitophorous vacuole. On the hepatocyte surface, CD81 and SR-B1 engage with P36 and P52 to facilitate rhoptry secretion at the sporozoite’s apical side. CD81 and SR-B1 are essential for hepatocyte infection by *P. falciparum* and *P. vivax*, respectively. Thus, critical components for successful sporozoite invasion and infection in liver organoids include: (1) HSPGs for sporozoite adhesion and (2) CD81 and SR-B1 for species-specific hepatocyte infection. However, hepatic organoids are often embedded in Matrigel, an extracellular matrix-based gel. This gel may act as a physical barrier that impedes direct sporozoite contact with hepatocytes. One potential solution is to use scaffold-free organoid platforms or apply the gel above (gel-topping) rather than embedding cells within it. Alternatively, dissociating the organoid into single cells may permit direct sporozoite-hepatocyte interactions. Following dissociation, the cells can be plated in a 2D culture or reassembled into a 3D organoid.

### 5.2 Post-sporozoite infection

Studies by Chua and colleagues and Rao and colleagues [62,69] confirm that 3D spheroid or organoid platforms can model liver-stage malaria. Yet, two major limitations persist. The first involves assessing complete parasite development where sporozoites form parasitophorous vacuoles multiply and release viable merozoites. Detecting these stages commonly relies on immunofluorescence, which often requires preparing cross-sections of spheroids or organoids and incubating them with specific antibodies. This approach only samples a portion of the culture, and low infection rates further complicate detection.

One attempt to improve detection involves dissociating hepatic spheroids and replating them as a monolayer on collagen before performing antibody-based assays. This method yields an overall infection rate but adds multiple steps, reducing the feasibility of high-throughput drug or compound screening. Unlike conventional 2D primary human hepatocyte cultures, high-content cell imaging can provide unbiased, highly sensitive quantification and staging of infecting sporozoites [40]. Another challenge is hypnozoite formation and reactivation. The hepatic organoid must remain functional for at least 10 days to support hypnozoite formation. Current protocols for hepatic organoid generation maintain hepatocyte functions for only about 3–27 days [47,66,72,81–87], limiting the study of dormant stages.

## 6. Challenges in the detection of hypnozoites

Modeling hypnozoite formation and reactivation is more complex than modeling schizogony. Rodent malaria parasites do not form hypnozoites, and simian malaria models, while informative, are costly and require highly regulated facilities. Historically, hypnozoites were first identified in *P. cynomolgi*, a simian parasite. In infected rhesus monkeys, an approximately 5 µm intracellular form persisted in liver sections at 7 days post-infection and remained unchanged at 105 days [88]. Whether these small forms eventually reactivate to cause blood-stage malaria remains unknown.

To date, hypnozoites are mainly inferred by parasite size and the time elapsed post-infection. In 2018, a recombinant monoclonal antibody against *P. vivax* UIS4 became available, enabling detection of the parasitophorous vacuole membrane [89]. A small, stable UIS4-positive vacuole now serves as a surrogate marker of hypnozoites [49]. Current *in vitro* models show evidence of hypnozoite reactivation. In micropatterned primary human hepatocyte co-cultures, Gural et al. observed spontaneous *P. vivax* hypnozoite reactivation, indicated by large schizonts emerging after the initial hypnozoite wave [38]. Using a transgenic *P. cynomolgi* line with a dual fluorescent reporter, Voorberg-van der Wel and colleagues showed that hypnozoites develop into schizonts in primary rhesus hepatocyte cultures [90]. Some studies suggest hypnozoite reactivation based on the disappearance of large forms and the persistence of small forms in human liver-chimeric mice and liver-on-chip systems [52,91]. Subsequently, the reappearance of large form schizonts implies that small forms may have reactivated. Both internal and external factors could influence hypnozoite activation, including geographic location, parasite strain characteristics, and host immunity [92]. Elucidating hypnozoite reactivation mechanisms demands a model that supports long-term culture and intervention testing.

## 7. Future perspective

Increasing ethical concerns about animal experimentation and improved cell culture methods have enhanced *in vitro* disease models. To replace existing models, future liver-stage malaria platforms must incorporate molecular and cellular components identified in prior studies. Findings from two-dimensional cell cultures and *in vivo* experiments will guide the design of three-dimensional (3D) models. Spheroid cultures improve cell functionality more than conventional two-dimensional hepatic cancer or immortalized cell lines. However, spheroids do not fully replicate native tissue microarchitecture. Organoids more closely mimic the structure and function of actual tissues. Yet, sporozoite invasion remains challenging due to gel-like extracellular barriers in organoid systems. Detecting schizonts and hypnozoites using antibodies in 3D models currently requires tissue-like analysis, producing only representative images. Employing transgenic *Plasmodium* strains with fluorescent reporters and whole-tissue analytical methods, such as tissue clearing, could improve infection quantification and treatment efficacy assessment. Advances in materials science may also enable microwell platforms with fluidic flow, further replicating the dynamic conditions of native tissues.
